# Quantitative global and gene-specific promoter methylation in relation to biological properties of neuroblastomas

**DOI:** 10.1186/1471-2350-13-83

**Published:** 2012-09-17

**Authors:** Nimrod B Kiss, Per Kogner, John Inge Johnsen, Tommy Martinsson, Catharina Larsson, Janos Geli

**Affiliations:** 1 Departments of Molecular Medicine and Surgery, Stockholm, Sweden; 2 Women’s and Children’s Health, Karolinska Institutet, Stockholm, Sweden; 3 Center for molecular medicine CMM, Karolinska University Hospital, Stockholm, Sweden; 4 Department of Biosciences, Sahlgrenska University Hospital, Göteborg, Sweden

**Keywords:** Neuroblastoma, Pyrosequencing, CIMP, BLU, CASP8, DCR2, CDH1, RASSF1A, RASSF2

## Abstract

**Background:**

In this study we aimed to quantify tumor suppressor gene (TSG) promoter methylation densities levels in primary neuroblastoma tumors and cell lines. A subset of these TSGs is associated with a CpG island methylator phenotype (CIMP) in other tumor types.

**Methods:**

The study panel consisted of 38 primary tumors, 7 established cell lines and 4 healthy references. Promoter methylation was determined by bisulphate Pyrosequencing for 14 TSGs; and *LINE-1* repeat element methylation was used as an indicator of global methylation levels.

**Results:**

Overall mean TSG Z-scores were significantly increased in cases with adverse outcome, but were unrelated to global *LINE-1* methylation. CIMP with hypermethylation of three or more gene promoters was observed in 6/38 tumors and 7/7 cell lines. Hypermethylation of one or more TSG (comprising TSGs *BLU*, *CASP8*, *DCR2*, *CDH1*, *RASSF1A* and RASSF2) was evident in 30/38 tumors. By contrast only very low levels of promoter methylation were recorded for *APC*, *DAPK1*, *NORE1A*, *P14*, *P16*, *TP73*, *PTEN* and *RARB*. Similar involvements of methylation instability were revealed between cell line models and neuroblastoma tumors. Separate analysis of two proposed *CASP8* regulatory regions revealed frequent and significant involvement of CpG sites between exon 4 and 5, but modest involvement of the exon 1 region.

**Conclusions/significance:**

The results highlight the involvement of TSG methylation instability in neuroblastoma tumors and cell lines using quantitative methods, support the use of DNA methylation analyses as a prognostic tool for this tumor type, and underscore the relevance of developing demethylating therapies for its treatment.

## Background

Neuroblastomas and ganglioneuromas are childhood tumors of the sympathetic nervous system that develop from primitive, neural crest-derived cells similar to those of the adrenal medulla and the sympathetic ganglia [1-3]. Afflicted patients present highly variable clinical courses, with spontaneous regression or critical tumor progression as two extreme and contrasting outcomes [2]. A number of clinical features and tumor phenotypes influence the disease outcome; the most important are age at diagnosis, tumor stage, amplification of the *MYCN* oncogene, activating *ALK* mutations, and somatic loss within chromosomal region 1p [1,2,4-8]. More recently, hypermethylation of tumor suppressor gene promoters were shown to be frequent in neuroblastoma with possible prognostic implications [9,10].

DNA methylation most commonly refers to methylation of the C in a CpG dinucleotide motif; a modification of fundamental importance for epigenetic regulation [11]. In normal cells, DNA methylation is central in processes such as expressional regulation, parental imprinting, and X-chromosome inactivation in females [11]. Abundant CpG methylation in promoter regions is widely associated with epigenetic silencing of gene transcription [11]. Aberrant DNA methylation is increasingly observed in various diseases. In cancer, promoter hypermethylation may be an alternative mechanism of tumor suppressor gene (TSG) silencing, which is otherwise associated with genetic mechanisms like mutation and deletion [12-14]. Indeed, concerted anomalous hypermethylation of TSG promoters is reported in an increasing number of cancer types, including among others neuroblastomas [10,15-18], and has been termed CpG island methylator phenotype (CIMP) [19]. Several reports indicate an association between the CIMP phenotype, advanced tumor disease and adverse outcome [15,16]. However, it is presently unknown whether increasing TSG hypermethylation is selected for during progression [16], or if aberrant DNA hypermethylation triggered by unknown factors confer epigenetic changes responsible for tumor progression.

The present view holds that cancer cells carry localized promoter hypermethylation together with global hypomethylation [20]. The *LINE-1* group of retrotransposon elements comprises approximately 17% of the human genome [21] and has been used as an analogue for genome-wide DNA methylation levels [21-26]. The *LINE*-1 retrotransposon promoter element is frequently hypomethylated in cancers [24,26]. The relationship between CIMP and *LINE-1* methylation is presently unknown, however a recent study suggested that *LINE-1* hypomethylation is inversely correlated with CIMP in colorectal cancer [27].

While several studies have assessed gene methylation in neuroblastomas, the methods employed were nonquantitative [9,10,28,29]. The present study was undertaken to quantitatively assess alterations of CpG methylation globally and in 14 prominent TSG promoters in relation to pathologic phenotypes in neuroblastomas.

## Results

### Hypermethylation of BLU, CASP8, DCR2, CDH1, RASSF1A and RASSF2 in neuroblastoma tumors and cell lines

CpG methylation status was assessed for regulatory regions of 14 tumor suppressor genes in 38 tumors and 4 healthy adrenal medullary reference samples (Table 1). For *CASP8* two alternative regulatory regions were analyzed, referred to as *CASP8* A1 and *CASP8* A2, and based on the findings *CASP8* A1 was selected for the further analyzes. Several of the investigated TSGs have chromosomal locations that are recurrently lost in neuroblastoma [30,31]: TP73 on 1p, BLU, RARB and RASSF1A on 3p, and PTEN on 10q. Epigenetic silencing of these genes would account for the second hit in Knudson’s 2-hit hypothesis. As expected, the majority of genes showed negligible or very low mean levels of methylation (below 10%) in the reference samples, and the cut-off for hypermethylation was therefore set to >10% (Table 1). *DCR2*, *RASSF1A* and *CASP8A2* displayed higher levels of intrinsic methylation in reference samples, and consequently cut-offs for hypermethylation were set at 30%, 30% and 50% respectively. In the tumor panel, increased promoter methylation was observed in *BLU*, *CASP8*, *DCR2*, *CDH1*, *RASSF1A* and *RASSF2* (Table 1), possibly providing the second hit for BLU, RARB and RASSF1A. An increase in methylation compared to reference samples was especially prominent in *RASSF1A* (> 60% of the tumors) and *CASP8* A1 (> 50% of the tumors). By contrast hypermethylation was not observed for *APC*, *DAPK1*, *NORE1A*, *P14*, *P16*, *TP73*, *PTEN* or *RARB* (Table 1).

**Table 1:** Overview of methylation levels in normals and primary tumors

	**No. of**	**Normals (% met)**		**Hypermethylation**	**Tumors (% met)**		**Tumors > cut-off**	
	**CpGs**	**mean**	**range**	**cut-off (% met)**	**mean**	**range**	**NB**	**Gang**
*Genes with detected hypermethylation*								
*BLU*	8	4	(3-4)	>10	7	(2-65)	4	0
*CASP8 - A1*	4	1	(0-3)	>10	16	(0-52)	21	0
*CASP8 - A2*	5	36	(24-41)	>50	42	(15-64)	9	0
*DCR2*	9	18	(10-21)	>30	12	(0-67)	5	0
*CDH1*	9	4	(4-5)	>10	3	(1-12)	1	0
*RASSF1A*	5	13	(8-21)	>30	43	(1-89)	23	0
*RASSF2*	6	6	(4-8)	>10	6	(3-11)	1	0
*Genes without detected hypermethylation*								
*APC*	10	2	(1-4)	>10	1	(0-2)	0	0
*DAPK1*	13	1	(0-1)	>10	0	(0-1)	0	0
NORE1A*	13	1	(1-3)	>10	1	(0-3)	0	0
*P14*	13	5	(2-11)	>10	1	(0-3)	0	0
*P16*	4	1	(0-1)	>10	0	(0-1)	0	0
*TP73*	19	1	(1-2)	>10	1	(0-2)	0	0
*PTEN*	10	1	(1-1)	>10	1	(0-1)	0	0
*RARB*	10	6	(5-7)	>10	2	(0-5)	0	0
*Global methylation*								
*LINE-1*	multiple	67.7	(65.6-70.3)	-	64.1	(53.9-74.8)		

NB = Neuroblastoma; Gang = Ganglioneuroma.

* previously published in [32]. (Geli et al. 2008).

In the seven neuroblastoma cell-lines analyzed hypermethylation was present in *BLU*, *CASP8*, *DCR2*, *CDH1*, *RASSF1A* and *RASSF2*, but was not observed for *APC*, *DAPK1*, *NORE1A*, *P14*, *P16*, *TP73*, *PTEN* or *RARB* (Table 2). Hence, the overall methylation pattern in cell lines mirrored that in tumors, *i.e.* genes that were highly methylated in cell lines also featured hypermethylation in primary tumors (Table 2).

**Table 2:** Methylation levels in neuroblastoma cell lines compared to primary tumors

**Sample** **group**	***APC*** **(% met)**	***BLU*** **(%)**	***CASP8-*** **A1 (%)**	***CASP8-*** **A2 (%)**	***DAPK*** **(%)**	***DCR2*** **(%)**	***CDH1*** **(%)**	***NORE1A*** **(%)**	***P14*** **(%)**	***P16*** **(%)**	***TP73*** **(%)**	***PTEN*** **(%)**	***RARB*** **(%)**	***RASSF1A*** **(%)**	***RASSF2*** **(%)**
*Cut-off*															
Hypermethylation	>10	>10	>10	>50	>10	>30	>10	>10	>10	>10	>10	>10	>10	>30	>10
*NB Cell lines*															
IMR-32	2	6	**77**	**81**	0	**80**	9	3	5	0	1	1	-	**93**	6
SH-5YSY	1	**36**	**89**	**85**	0	**88**	**19**	2	1	1	1	1	-	**94**	**11**
SK-N-AS	1	-	0	**77**	0	**81**	6	3	2	0	5	1	-	**94**	**26**
SK-N-BE	-	-	**94**	-	-	**78**	-	-	-	1	-	0	-	**95**	-
SK-N-DZ	3	-	**42**	39	1	**37**	**12**	6	3	1	3	1	4	**92**	6
SK-N-F1	1	-	**50**	**72**	0	**76**	10	1	2	1	2	1	8	**95**	10
SK-N-SH	0	-	**78**	**80**	0	**86**	8	2	-	1	1	1	3	**93**	8
All	1	**21**	**61**	**73**	0	**75**	**11**	3	3	1	2	1	5	**94**	**11**
*NB Tumors*															
All	1	7	**16**	42	0	12	3	1	1	0	1	1	2	**43**	6
Number > cut-off	n = 0	**n = 4**	**n = 21**	**n = 9**	n = 0	**n = 4**	**n = 1**	n = 0	n = 0	n = 0	n = 0	n = 0	n = 0	**n = 23**	**n = 1**

met = methylation; Bold indicate hypermethylation.

**Figure 1:**
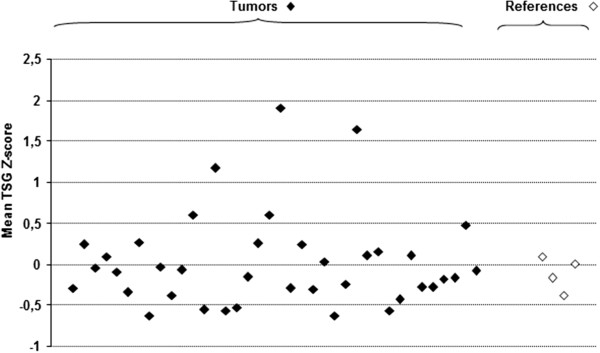
Comparison of mean TSG Z-scores between primary tumors (filled diamonds), and reference adrenal medullas (open diamonds).

For comparison of methylation densities Z-scores were calculated for each TSG as well as a mean for all TSG analyzed. Taking the whole tumor population into account there was no apparent statistically significant difference between tumors and references with regards to mean Z-scores (Figure 1).

**Figure 2:**
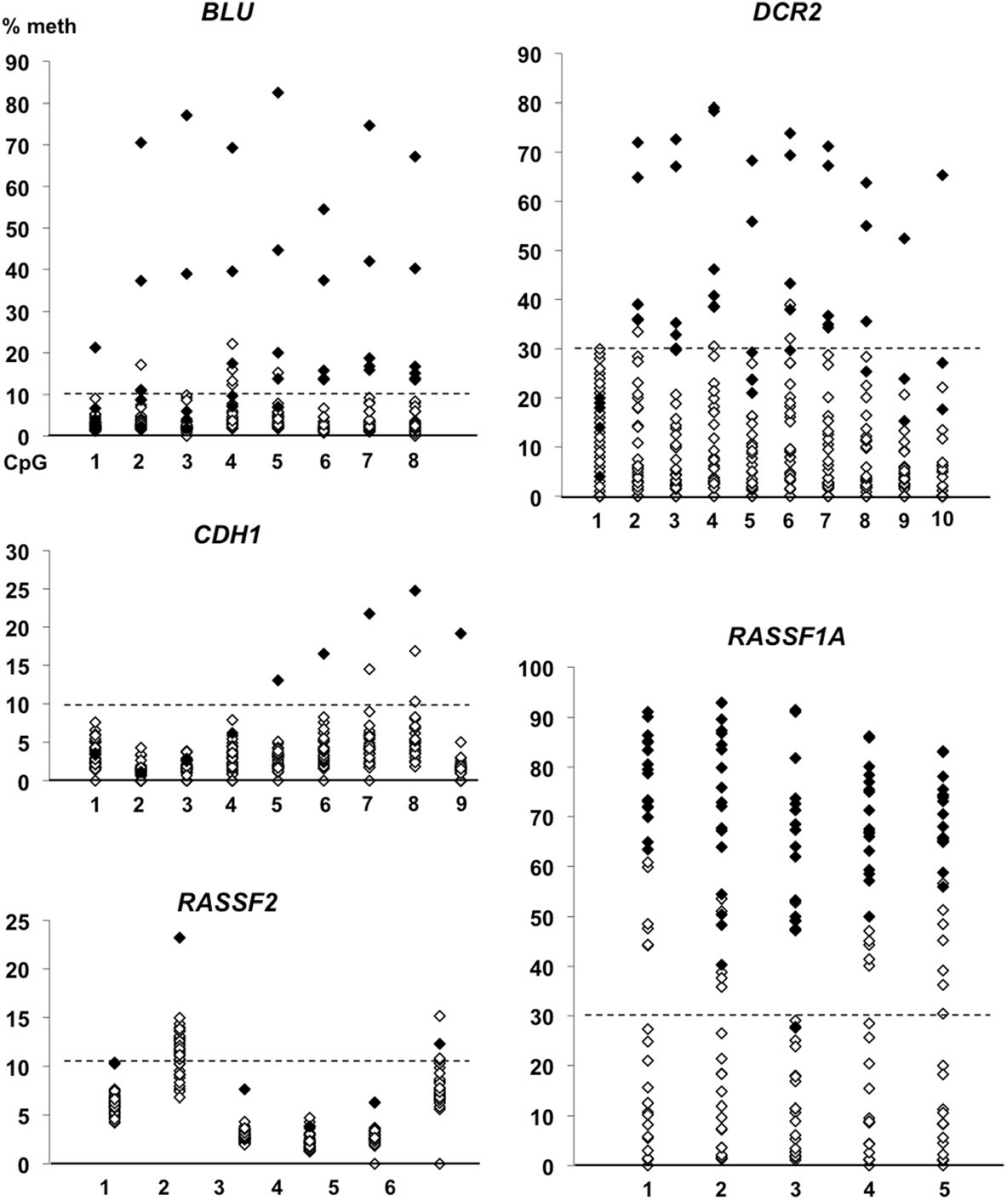
**Hypermethylation of promoter regions for *BLU, DCR2, CDH1, RASSF2 *and *RASSF1A *in primary tumors.** The scatterplots illustrate the distribution of methylation densities detected at each CpG analyzed in the individual tumors. Filled diamonds represent tumors with hypermethylation (the mean value for all CpGs of the gene are above cut-off), while tumors without hypermethylation are indicated as open diamonds. The cut-off levels for hypermethylation are marked by a dotted line for *BLU* (>10 %)*, CDH1* (>10 %), *RASSF2* (>10 %), *DCR2* (>30 %), and *RASSF1A* (>30 %).

**Figure 3:**
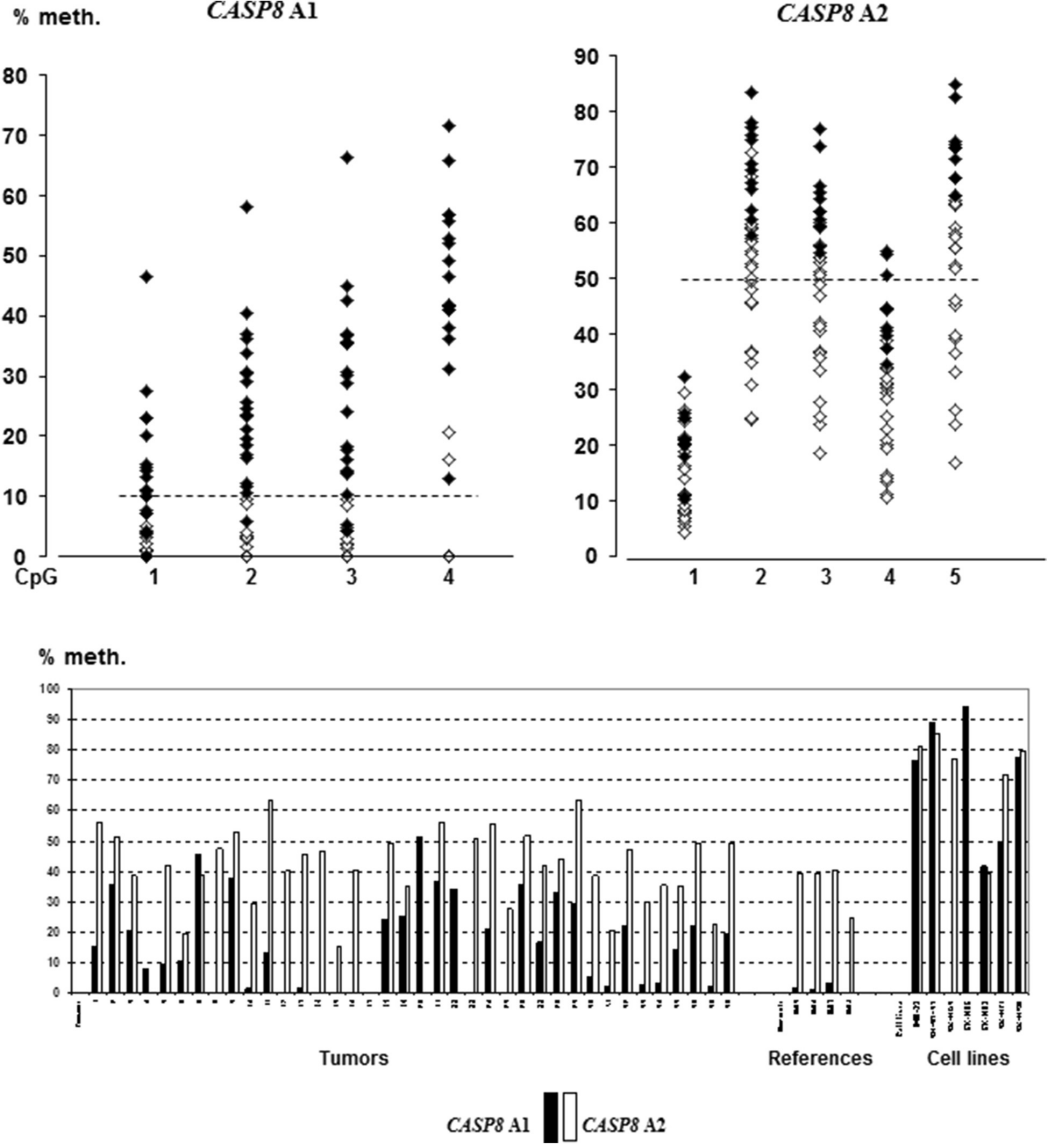
**CpG hypermethylation at the two reported regulatory regions of the *CASP8 *gene.** Methylation densities detected at the CpGs assayed in the respective locations *CASP8* A1 and *CASP8* A2 are shown at the top, where filled diamonds represent hypermethylated tumors and open diamonds indicate unmethylated tumors. The cut-off levels >10 % for *CASP8* A1 and >50 % for *CASP8* A2 are marked by dotted lines. The diagram at the bottom illustrates the relationship between mean methylation levels of *CASP8* A1 (filled staples) and *CASP8* A2 (open staples) in all samples analyzed including tumors, references and cell lines.

Hypermethylation above the cut-off range was evident for one or more TSG promoters in 30 of the 38 tumors. Additional file 1: Table S1 and Additional file 2: Table S2 details the results for each TSG in the 38 individual tumors concerning mean methylation density of all CpG sites as well as the range of minimum to maximum values for the individual CpGs recorded. In all cases genes without detected hypermethylation exhibited mean values below the cut-off at 10%. With one single exception the maximum values recorded for any individual CpG were also below the cut-off at <10% (Additional file 2: Table S2). These observations suggest that the *APC*, *DAPK1*, *NORE1A*, *P14*, *P16*, *TP73*, *PTEN* and *RARB* promoters are rarely methylated in neuroblastomas. Six genes showed promoter hypermethylation in primary tumors, and in addition, for some samples increased methylation was recorded at individual CpGs although the mean of all CpGs did not reach above the cut-off (Additioanal file 1: Table S1; Figures 2 and 3). *BLU* exhibited mean methylation >10% cut-off in 4 tumors and maximum methylation up to 22% in 10 additional tumors. Corresponding results were, for *CDH1* (1 tumor with mean >10% and 1 additional tumor with max at 17%), for *RASSF2* (1 tumor + 24 tumors up to 15%), for *DCR2* (5 tumors + 3 tumors up to 39%), for *RASSF1A* (23 tumors + 1 up to 31%), and for *CASP8* A1 (21 tumors + 2 tumors up to 21%). *BLU*, *CDH1*, *RASSF2*, *DCR2* and *RASSF1A* showed quite similar dispersal in methylation densities between individual CpGs (Figure 2). However for *CASP8* A1 a gradient of increased methylation was noted from CpG 1 to 2, 3, and 4 (Figure 3). Similarly, *CASP8* A2 showed the highest methylation at CpG 2, 3 and 5, moderate methylation at CpG 4 and the lowest levels at CpG 1 (Figure 3).

### CIMP phenotype in neuroblastoma tumors and cell lines

CIMP was here defined as tumors with concerted hypermethylation in 3 or more of the assessed TSGs in agreement with our previous definitions of this phenotype [32]. Six neuroblastomas, but no ganglioneuromas showed hypermethylation of 3 or 4 genes and thus met the criteria for CIMP (tumors 14, 18, 19, 20, 27 and 35; Table 3). All genes involved in hypermethylation in this study contributed to CIMP, and mean Z-scores and *DCR2* Z-scores were significantly higher in tumors classified as CIMP (p = <0.003). Similarly, all 7 cell-lines carried promoter hypermethylation for 3 to 6 genes in agreement with a CIMP phenotype (Table 2).

**Table 3:** Results for dysmethylated promoter regions in primary tumors

**Case**	**Primary tumor**			**High-risk**	**Follow**	***BLU***	***Casp8 A1***	***CASP8 A2***	***DCR2***	***CDH1***	***RASSF1A***	***RASSF2***	**Total**	**CIMP**	**Z-score**	***LINE-1***
**no.**	**type**	**stage**	***MYCN***	**therapy**	**up**	**>10%**	**>10%**	**>50%**	**>30%**	**>10%**	**>30%**	**>10%**	**no.**		**all genes**	**%**
1	NB	4S	-	-	NED	-	*CASP8 A1*	*+ CASP8 A2*	-	-	*RASSF1A*	-	2	-	- 0.30	64.8
2	NB	1	-	-	NED	-	*CASP8 A1*	*+ CASP8 A2*	-	-	*RASSF1A*	-	2	-	0.23	66.3
3	NB	3	-	-	NED	-	*CASP8 A1*	-	-	-	*RASSF1A*	-	2	-	- 0.04	58.0
4	NB	1	-	-	NED	-	-	-	*DCR2*	-	-	-	1	-	0.07	65.8
5	NB	4	yes	yes	DOD	-	-	-	-	-	-	-	-	-	- 0.11	68.2
6	NB	2A	-	-	NED	-	*CASP8 A1*	-	-	-	*RASSF1A*	-	2	-	- 0.33	55.7
7	NB	4	yes	yes	DOD	-	*CASP8 A1*	-	-	-	-	-	1	-	0.26	70.0
8	NB	2	-	-	NED	-	-	-	-	-	*RASSF1A*	-	1	-	- 0.65	58.2
9	NB	4	yes	yes	DOD	-	*CASP8 A1*	*+ CASP8 A2*	-	-	-	-	1	-	- 0.06	69.8
10	NB	4S	-	-	NED	-	-	-	-	-	-	-	-	-	- 0.37	62.9
11	NB	4	yes	yes	NED	-	*CASP8 A1*	*+ CASP8 A2*	-	-	-	-	1	-	- 0.06	66.4
12	NB	4	yes	yes	DOD	*BLU*	-	-	-	-	*RASSF1A*	-	2	-	0.57	62.4
13	Gang	-	-	-	NED	-	-	-	-	-	-	-	-	-	- 0.55	68.9
14	NB	3	yes	yes	NED	-	-	-	*DCR2*	-	*RASSF1A*	*RASSF2*	3	CIMP	1.19	57.0
15	Gang	-	-	-	NED	-	-	-	-	-	-	-	-	-	- 0.58	72.6
16	NB	4	yes	yes	NED	-	-	-	-	-	-	-	-	-	- 0.52	67.2
17	NB	2	-	-	NED	-	-	-	-	-	*RASSF1A*	-	1	-	- 0.14	57.1
18	NB	4	-	-	NED	-	*CASP8 A1*	-	*DCR2*	-	*RASSF1A*	-	3	CIMP	0.24	69.9
19	NB	3	-	-	NED	-	*CASP8 A1*	-	*DCR2*	-	*RASSF1A*	-	3	CIMP	0.55	59.7
20	NB	2	-	-	AWD	-	*CASP8 A1*	-	*DCR2*	*CDH1*	*RASSF1A*	-	4	CIMP	1.87	60.2
21	NB	4	yes	yes	NED	-	*CASP8 A1*	*+ CASP8 A2*	-	-	-	-	1	-	- 0.30	66.8
22	NB	2	-	-	NED	-	*CASP8 A1*	-	-	-	*RASSF1A*	-	2	-	0.21	55.6
23	NB	3	yes	yes	NED	*BLU*	-	*CASP8 A2*	-	-	-	-	2	-	- 0.32	65.9
24	NB	3	-	-	NED	-	*CASP8 A1*	*+ CASP8 A2*	-	-	*RASSF1A*	-	2	-	0.01	53.9
25	Gang	-	-	-	NED	-	-	-	-	-	-	-	-	-	. 0.62	65.9
26	NB	3	-	-	NED	-	*CASP8 A1*	*+ CASP8 A2*	-	-	-	-	2	-	- 0.25	59.7
27	NB	4	yes	yes	DOD	*BLU*	*CASP8 A1*	-	-	-	*RASSF1A*	-	3	CIMP	1.57	65.9
28	NB	4S	-	-	NED	-	*CASP8 A1*	-	-	-	*RASSF1A*	-	2	-	0.11	58.7
29	NB	3	-	-	DOC	-	*CASP8 A1*	*+ CASP8 A2*	-	-	*RASSF1A*	-	2	-	0.14	55.0
30	NB	1	-	-	DOC	-	-	-	-	-	*RASSF1A*	-	1	-	- 0.57	61.7
31	NB	1	-	-	NED	-	-	-	-	-	-	-	-	-	- 0.43	74.8
32	NB	2	-	-	DOD	-	*CASP8 A1*	-	-	-	*RASSF1A*	-	2	-	0.10	64.4
33	NB	1	-	-	NED	-	-	-	-	-	*RASSF1A*	-	1	-	- 0.28	67.7
34	NB	4	-	-	NED	-	-	-	-	-	-	-	-	-	- 0.29	71.5
35	NB	1	-	-	NED	*BLU*	*CASP8 A1*	-	-	-	*RASSF1A*	-	3	CIMP	- 0.20	67.9
36	NB	1	-	-	NED	-	*CASP8 A1*	-	-	-	*RASSF1A*	-	2	-	- 0.17	66.7
37	NB	3	yes	yes	NED	-	-	-	-	-	*RASSF1A*	-	1	-	0.48	66.0
38	NB	3	-	-	NED	-	*CASP8 A1*	-	-	-	*RASSF1A*	-	2	-	- 0.10	67.7

NB = neuroblastoma; AWD 0 Alive with disease; DOD = Dead of disease, NED = No evidence of disease; DOC = Dead of surgical complications.

### Analyses of alternative regulatory regions in CASP8

**Figure 4:**
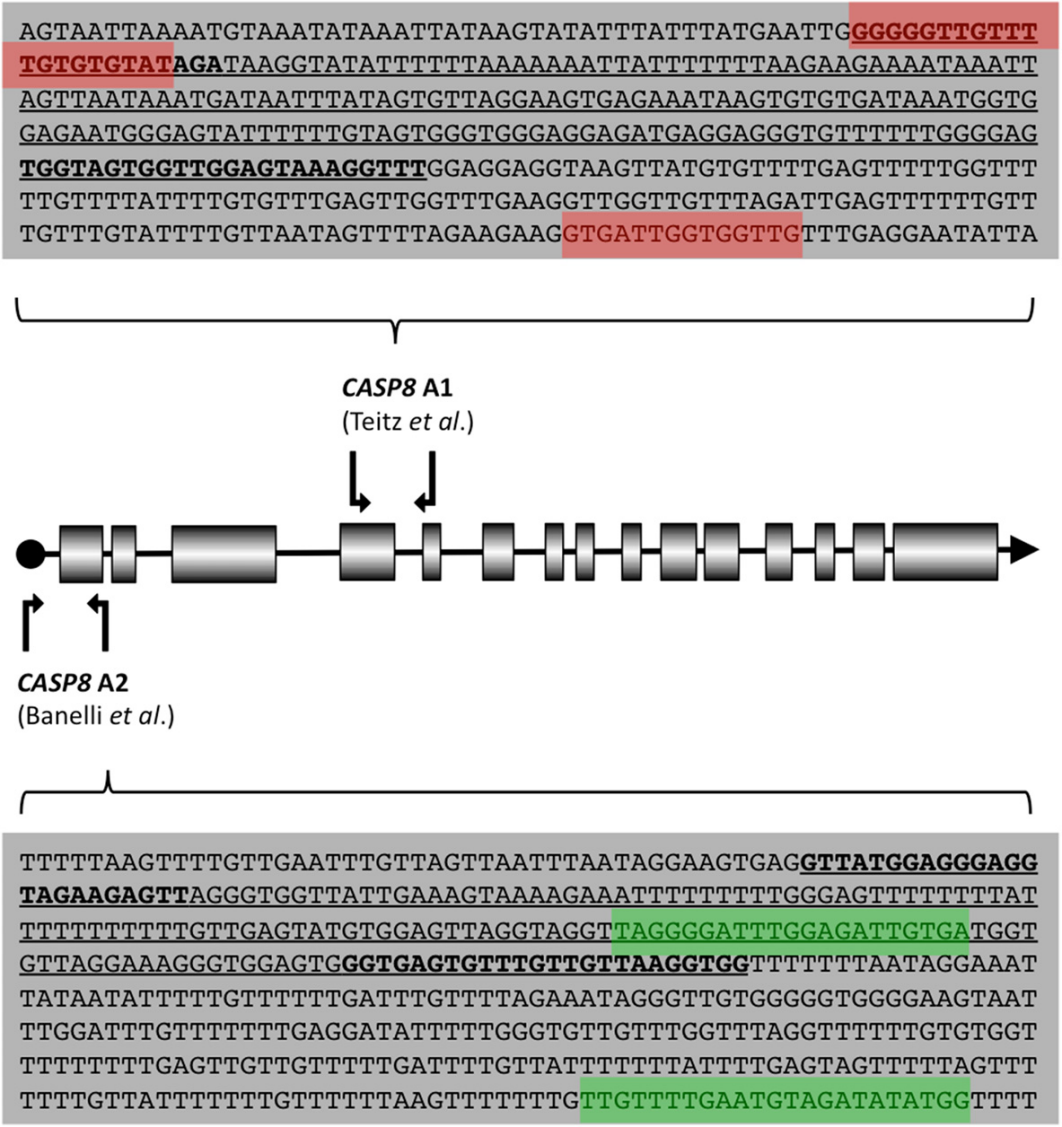
**Methylation analyzes of the two reported regulatory regions of the *CASP8 *gene .** The location of *CASP8* A1 [25] and *CASP8* A2 [27] are indicated in the schematic illustration of the *CASP8* gene locus. Location of MSP primers applied by Teitz *et al.*, and Banelli *et al.,* are indicated in colour in the sequences shown. The locations of primers used in the present study are marked in bold and the sequence is underlined.

The two reported regulatory regions of *CASP8* are illustrated in Figure 4, together with the location of primers applied in previous and the present study. In this study, the mean methylation levels for *CASP8* A2 were found to be high, between 15-64%, but this was mirrored by prominent methylation also in the reference samples (24-41%). Nine tumors reached a methylation level above the 50% cut-off level. In 8 of these 9 tumors hypermethylation was also observed for the *CASP8* A1 region (Table 3). For *CASP8* A1 methylation levels in reference adrenal medullas were low <10%, while hypermethylation above the cut-off was recorded in 21 tumors (Table 3).

### Comparison of TSG methylation with clinical and genetic features

**Figure 5:**
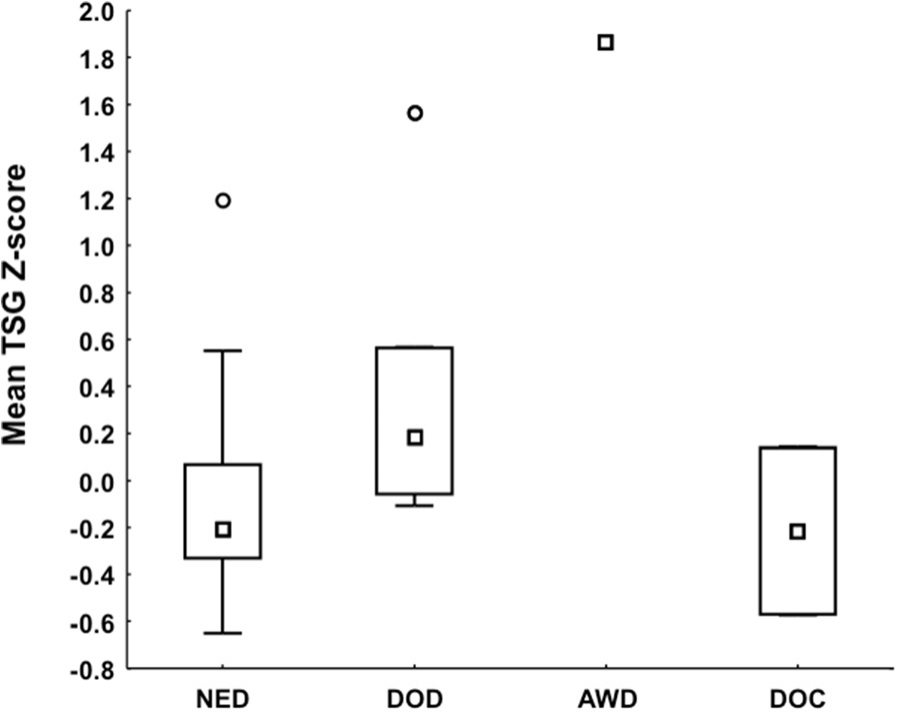
**Association between Z-score and patient outcome at follow-up.** Box-plots illustrate mean TSG Z-scores in patients with different outcome at follow-up; no evidence of disease (NED), dead of disease (DOD), alive with disease (AWD), or dead of surgical complications (DOC).

**Figure 6:**
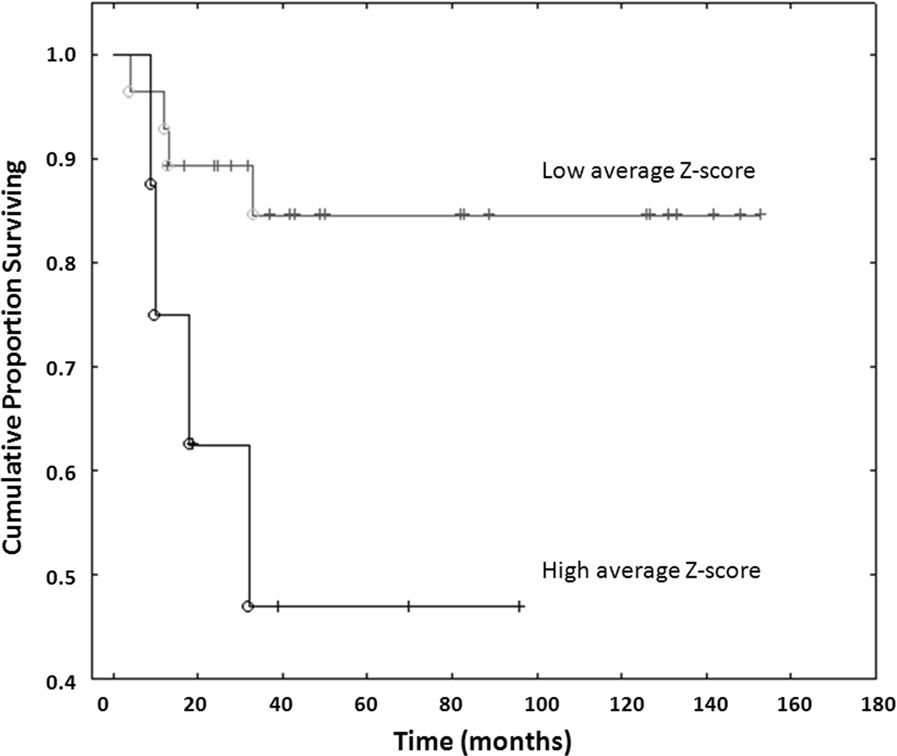
**Kaplan-Meier plots illustrating survival in patients with an average Z-score higher or lower than the median of the Z-scores for all methylated TSGs** (Table 3). There is a significant difference between the two groups (p = <0.04), indicating adverse outcome in the group with increased mean Z-scores.

Mean TSG Z-scores were significantly higher in tumors from patients with adverse outcome at follow-up *i.e.* patients who died of disease (DOD) or were alive with disease (AWD) *vs.* cases with no evidence of disease (NED) (Kruskal-Wallis p = 0.04; Figures 5 and 6). Mean TSG Z-score, *CASP8* A1 Z-score and *RASSF1A* Z-score were also significantly higher in neuroblastomas than in ganglioneuromas (Mann–Whitney *U* Test p-values =0.025, 0.014, <0.009 respectively).

As expected significant associations were apparent between tumor stage *vs. MYCN* amplification; *vs.* 1p loss; and *vs.* high-risk, as well as between *MYCN* amplification *vs.* 1p loss and *vs.* high risk. High-risk was also significantly correlated to poor outcome. The strong correlation between these features support the representativity and relevance of the material under study.

### Global LINE-1 methylation

**Figure 7:**
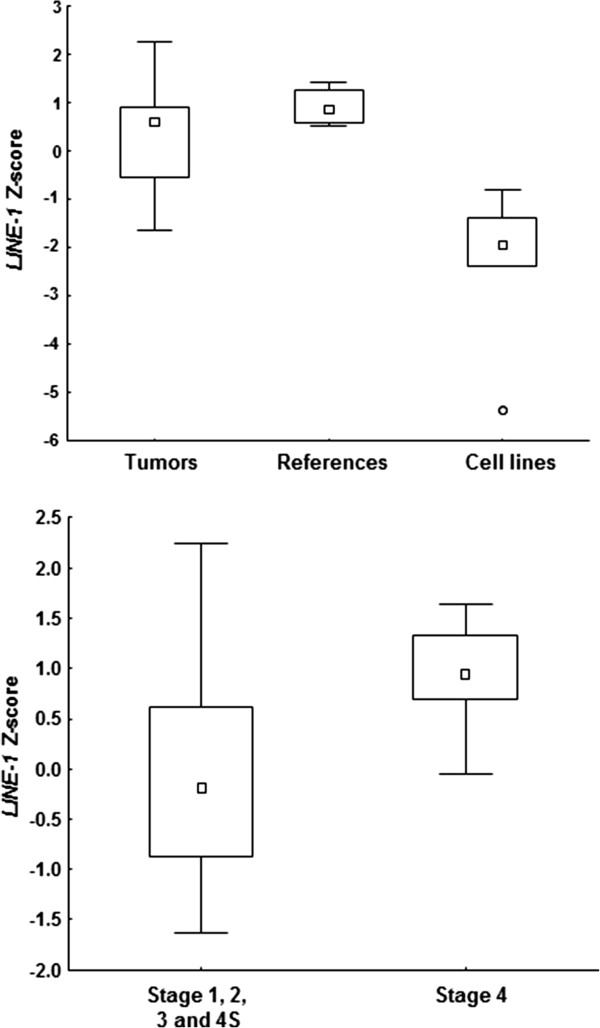
**Global methylation levels of *LINE-1 *repeats in the different sample groups, and association between *LINE-1 *and tumor stage.** Box-plots at the top show Z-scores for *LINE-1* repeat elements in primary tumors, reference adrenal medullas and neuroblastoma cell lines. Box-plots below illustrate relatively higher *LINE-1* Z-scores in Stage 4 as compared to Stage 1, 2, 3 and 4 S tumors.

Methylation density of *LINE-1* repeat elements was determined as a measure of the global methylation status. High and comparable levels of *LINE-1* methylation were recorded in reference samples and primary tumors, while the levels were lower in neuroblastoma cell lines (Figure 7). *LINE-1* Z-scores were not significantly different between tumors and reference adrenal medulla. There was a negative correlation between *LINE-1* and *RASSF1A* Z-scores (Spearman Rank Order Correlation −0.56; p < 0.05). Stage 4 neuroblastomas showed higher *LINE-1* methylation *vs.* lower stage tumors (Mann–Whitney *U* Test; p = 0.01; Figure 7). *LINE-1* methylation was not correlated to CIMP or other tumor features.

## Discussion

In the current study we used quantitative bisulfite Pyrosequencing to assess promoter methylation levels of tumor suppressor genes known to be frequently hypermethylated in cancer. In 30/38 tumors we found significant hypermethylation in one or more of the following genes; *BLU*, *CASP8*, *DCR2*, *CDH1*, *RASSF1A* and *RASSF2*. Overall Z-scores for the TSGs assessed were significantly associated with adverse outcome. Furthermore, six of the 38 tumors conformed to the criteria for CIMP, *i.e.* CpG island methylator phenotype. The non-tumoral adrenal medullary material in this study represents the closest possible healthy analogue to the cells that comprise neuroblastomas. Neuroblastomas arise solely in neural crest-derived cells of sympathoadrenal lineage ( [33] and others), the very cells that would form the adrenal medulla (and abdominal sympathetic ganglia) in healthy individuals [3]. In this current study they were only used to confirm that TSG methylation is low in healthy tissue, and to determine suitable cut-off levels for different TSGs. The level of cut-off for hypermethylation in individual genes was set conservatively to exceed the methylation density observed in the reference adrenal medulla (Table 1). For genes where no methylation was observed the cut-off was set to 10% to avoid false positives resulting from background fluctuation. For the genes where methylation was detected in reference adrenal medulla (*CASP8A2*, *DCR2*, *RASSF1A*) the cut-off in tumors was set well above the level observed in the reference samples (Table 1). Other recent publications on DNA hypermethylation in neuroblastoma either do not utilize reference controls [9,10], or compare to “normal adrenal” and blood lymphocyte DNA [34]. We used the reference tissue only to assess presence or absence of hypermethylation; for the comparison between methylation levels and clinical/genetic phenotypes, we took into account Z-scores for all 38 tumors in the series, independent of whether they were classified as hypermethylated or not. Hence, methylation levels in reference adrenal medullary material were not included in any statistical calculation.

Although several recent studies have assessed methylation in genes with putative tumor suppressor properties [9,10], this current study is the first to employ a quantitative method, pyrosequencing, to assess promoter methylation in multiple TSGs. Our data partially corroborate the findings of Grau *et al.* and Hoebeeck *et al.*, as we find abundant methylation in *CASP8A* and *RASSF1A*; however, we did not observe significant hypermethylation in the *PTEN* promoter as reported by Hoebeeck *et al.* We here acknowledge the sensitivity of methylation specific PCR (MSP) in detecting low levels of methylation. However, this highly sensitive, nonquantitative assay is known to produce false positive results [35,36]. Further, the biological significance of methylation detected by MSP may be limited, as the technique is capable of producing positive results down to a methylation level <1% (Rand *et al.*, own observations). This corresponds to a very limited subset of cancerous cells in a tumor, and may in fact represent DNA methylation in contaminating cell types. This underscores the importance of employing quantitative methods when assessing DNA methylation – whenever possible combined with relevant reference samples for the sake of arbitration.

*CASP8*, defined as a tumor suppressor gene by Teitz *et al.*[28], promotes apoptosis upon activation of the Fas apoptotic pathway through the Fas ligand [37]. There has been some debate concerning the localization of the *CASP8* regulatory region. In 2000 Teitz *et al.* reported agreement between methylation of a CpG-rich region (defined by GenBank accession number AF210257, positions 536–856; Figure 4) and absent *CASP8* expression in neuroblastomas. Also, the region was reported to be methylated almost exclusively in neuroblastomas with *MYCN* amplification. Teitz *et al.* indicated that this could signify that inactivation of the Fas apoptotic pathway is needed for the survival of neuroblastoma cells expressing high levels of *MYCN*. A contrasting view was presented by Banelli *et al.* who did not find correlation between *CASP8* silencing and *MYCN* amplification, although higher frequencies of methylation were detected in *MYCN*-amplified cells [29]. They also argued that the CpG-rich intragenic region assessed by Teitz *et al.* is not a true regulatory region for *CASP8*. Instead, a region flanking exon 1 was proposed as the *CASP8* promoter (Figure 4). In the current paper we have assessed the regulatory regions suggested in both publications, designated as *CASP8* A1 [28] and *CASP8* A2 [29] (Figure 4). Furthermore, we have compared the methylation levels in these sites in neuroblastomas to those of healthy reference tissues, which was not undertaken in the previous studies. The differences observed between methylation of the regions *CASP8* A1 and *CASP8* A2 in reference adrenal medullary DNA are striking: the region *CASP8* A1, while abundantly methylated in neuroblastomas (mean methylation 16.2%; 21 tumors over cut-off), was devoid of methylation in reference adrenal medulla. In contrast, *CASP8* A2 had a high degree of methylation in both tumors and references(9 tumors over cut-off; mean methylation in tumors 42% *vs.* 36% in reference samples). Thus, hypermethylation at *CASP8* A1 was found as a better indicator of a pathologic condition than high methylation at the *CASP8* A2 region. Furthermore, *CASP8* A1 and *CASP8* A2 both showed striking variations in methylation densities between individual CpGs (Figure 3). For *CASP8* A1 a gradient with increasing methylation from CpG 1 to CpG 4 was noted, while for *CASP8 A2* very high methylation was frequently recorded at CpG 2, 3 and 5 (Figure 3). These observations underline the importance of analyzing more than single CpGs as an indicator of methylation density.

A long-standing dilemma in neuroblastoma research is the proposed association between *CASP8* methylation and *MYCN* amplification [9,10,28,38]. Like Grau *et al.* we do not find a correlation between *CASP8 A1* methylation and *MYCN* amplification [10]. In contrast, Hoebeeck *et al.*, who also assessed the *CASP8 A1* region, performed a meta-analysis including a total of 115 neuroblastomas that linked methylation of the *CASP8 A1* region with *MYCN*-amplification [9]. However, the included studies utilized non-quantitative MSP [9,28,38]. Our findings provide an epigenetic explanation to a previous study wherein loss of CASP8 protein expression was observed in a majority of neuroblastomas [39]. We further support, backed by quantitative epigenetic data, the observation from that study that no correlation exists between loss of CASP8 and adverse neuroblastoma features and outcomes (such as *MYCN* amplification and reduced survival) [39].

The CpG Island Methylator Phenotype, CIMP, is characterized by concerted abnormal hypermethylation in CpG rich gene promoters. Such epigenetic remodeling could lead to the simultaneous inactivation of cellular functions that regulate growth, differentiation and apoptosis and thus contribute to neoplasia development and disease phenotype. Indeed, CIMP has been described in a number of cancers, including neuroblastomas, often in conjunction with unfavorable disease progression [15,16]. Abe *et al.* defined CIMP as simultaneous methylation in CpG islands of the *PCDHB* and *PCDHA* gene families, and the *HLP*, *DKFZp451I127* and *CYP26C1* genes, and found this genotype associated to *MYCN* amplification [17,18]. In the current study CIMP was observed in 6/38 tumors and in 7/7 cell lines. However, no significant correlations between CIMP and clinical/genetic features were observed. These results support that concerted promoter hypermethylation is an important facet of the neuroblastoma causality, however if hypermethylation of key TSGs are involved in fatal disease progression they would partly differ from those assessed in this study. By contrast, significantly higher mean TSG Z-scores were observed in tumors with poor outcome at follow-up, which further indicates that hypermethylation is a component of morbidity in neuroblastomas. Analysis of a subset of TSG promoters is likely to render a somewhat fragmental insight into the role of promoter hypermethylation in neuroblastoma development. More global approaches such as methylation arrays are likely to yield a more detailed account of the key genes which, by undergoing hypermethylation, significantly impact tumor progression.

*LINE-1* is frequently hypomethylated in cancer [24,26], leading to its activation – which in turn causes genomic instability [40]. In the current study *LINE-1* Z-scores were higher in stage 4 tumors as compared to tumors of stage 1, 2, 3, and 4 S, indicating global hypermethylation. The finding indicates that genomic instability in metastatic neuroblastomas is not caused by *LINE-1* activation. Taking into consideration that DNA methylation is a disseminative event, this finding contributes to an emerging picture of aberrant epigenetic patterning in deleterious neuroblastomas.

Several TSGs showed prominent promoter hypermethylation in this study. The two most notably methylated TSG promoters were *CASP8* A1 and *RASSF1A*. Furthermore *BLU* and *DCR2* also exhibited high levels of methylation, and have been reported previously in association with CIMP [17]. Interestingly, serum levels of *RASSF1A* and *DCR2* methylation were reported to have prognostic importance [41,42]. The epigenetic inactivation of genes with such diverse tumor suppressive functions as growth arrest (*RASSF1A*) and apoptosis (*CASP8* A1 and *DCR2*) may be requisite for neuroblastoma tumorigenesis. In this regard it is interesting to observe that promoter hypermethylation in the same genes occurred in neuroblastoma cell lines, a phenomenon also observed by Hoebeeck *et al.*[9]. In the current study the relative methylation patterns were similar between cell lines and primary tumors (Table 2): for TSGs in which high levels of methylation were observed in cell lines, methylation was abundant even amongst the tumor samples. An appealing prospect from this is that neuroblastoma cell lines may be scanned for putative methylation in TSGs, perhaps using array techniques, in order to identify the most relevant TSGs in neuroblastomas. In an approach by Carén *et al.*[34] that utilized chemical de-methylation of cell lines in combination with expression arrays a number of genes were identified as differentially methylated in neuroblastomas. Interestingly, no *bona fide* TSGs were identified. Several studies now confirm the presence of gene hypermethylation in neuroblastoma. While this is an important observation the cause-effect relationship between tumorous neuroblastoma and gene hypermethylation should be further investigated to facilitate improved treatment approaches.

## Conclusions

This study provides a quantitative corroboration of previous observations that DNA methylation, in a subset of tumor suppressor genes, is a common event in neuroblastomas; and that it is associated with adverse outcome of the disease. The findings demonstrate a comparable involvement of methylation instability in neuroblastoma tumors and neuroblastoma models, and support an advancement of therapeutic strategies that include the use of demethylating agents to counter TSG silencing in this tumor type.

## Materials and methods

### Ethics statement

The thirty-eight samples of neuroblastoma/ganglioneuroma studied were obtained from patients operated at the Karolinska University Hospital. All samples were initially collected with informed verbal consent from patients or their legal guardians as documented in the patient's medical journal. The collection and subsequent study of the tissue material has been approved by the Karolinska Institutet/Karolinska University Hospital Research Ethics Committee.

### Cell lines

Seven neuroblastoma cell lines were included in the study: SK-N-DZ, SK-N-SH, SK-N-BE, SK-N-FI, SK-N-AS, IMR-32, and SH-SY-5Y. Cells were grown as previously described [43].

### Patients and tumor samples

A total of 38 primary tumors from 38 patients were studied (case no 1–38). Clinical details and tumor characteristics have been previously published for all cases [44]. The tumor panel includes 35 neuroblastomas and 3 ganglioneuromas diagnosed between 0 m and 145 m of age. Information for *MYCN* amplification and 1p loss have been previously reported [44]. Results for *NORE1A* methylation in tumors have been previously published [44].

### Non-tumor controls

DNA from histopathologically evaluated non-tumoral, healthy adrenal medulla (N1-N4) was acquired from Clinomics Biosciences, Inc. (Watervliet, NY, USA) [45]. The neural crest-derived cells of this healthy adrenal medullary material came from adult individuals, and represents the closest possible analogy to the neural crest-derived cells that comprise neuroblastomas. For the validation of individual pyrosequencing assays methylated human DNA was purchased from Millipore/Chemicon (Billerica, MA, USA) and used as positive control, while normal lymphocyte DNA served as negative control. Serial dilutions were made between *in vitro* methylated DNA and unmethylated DNA and analyzed by Pyrosequencing to assess potential PCR bias towards either the methylated or the unmethylated form. This was done for all in-house designed Pyrosequencing assays (Additional file 3: Table S3) employing ratios of 100%, 75% 50% 25% and 0% of methylated *vs.* unmethylated DNA.

### Extraction of DNA

Tissues were immediately snap frozen in liquid nitrogen upon surgical removal. DNA extraction from primary tumor samples and neuroblastoma cell lines was carried out either by means of a standard phenol-chloroform purification procedure or by applying the ChargeSwitch gDNA Mini Tissue Kit (Invitrogen/Life Technologies Corporation, Carlsbad, CA). A NanoDrop Spectrophotometer (ND-1000) was used to quantify the DNA.

### Detection of promoter methylation

Promoter methylation was quantified by Pyrosequencing for 14 TSGs in tumors, cell-lines, reference samples, and controls. 500 ng of each DNA sample was bisulfite treated using the EZ DNA Methylation Gold Kit (Zymo Research, Orange, CA, USA) and 25–50 ng was subsequently amplified in each gene-specific PCR reaction containing the following reagents: 0.2 mM of each primer (Additional file 3: Table S3), 0.2 mM dNTPs, 1.6 units of HotStarTaq, and 10x PCR buffer (QIAGEN) in a final volume of 50 μl. The reaction for *DCR2* contained additional MgCl_2_ (QIAGEN) in a final concentration of 3.0 mM. PCR conditions were: 95°C for 15 minutes then cycled 45 times at 95°C for 20 seconds, a gene-specific annealing temperature (Additional file 3: Table S3) for 20 seconds and 72°C for 20 seconds, followed by an extension at 72°C for 10 minutes. PCR was followed by Pyrosequencing using specific sequencing primers (Additional file 3: Table S3) in either a Biotage PSQ^TM^ 96MA Pyrosequencer or in a Biotage PyroMark^TM^ Q24 Pyrosequencer. Methylation of *LINE-1* repeat elements were analyzed as a measurement of global methylation levels using previously described methodology [24]. The standard operating procedure for performing pyrosequencing includes an internal control against incomplete bisulfite conversion: The nucleotide dispensation order is programmed in such a way that C nucleotides are dispensed where the sequence contains a non-CpG cytosine. If the bisulfite treatment is consummate, all of these Cs have been converted to Ts, and there is no incorporation of the dispensed C nucleotide. Our assays contained such internal controls, and accordingly full bisulfite conversion was ascertained in all samples.

### Statistical analysis

All calculations were performed in Microsoft Excel 2003 SP3 and in the STATISTICA data analysis software, v. 7.0. P values ≤ 0.05 were considered significant. Only genes that displayed methylation above the cut-off levels were considered for statistical analysis. To gauge methylation as a continuous variable in multiple promoters, Z-scores were calculated for each gene according to the formula: (Mean of the CpG methylation density of the given promoter for each sample – mean of methylation density for that promoter in the tumor panel)/SD of that methylation density. A mean between Z-scores for all assessed TSG promoters was calculated for each sample, giving an overall methylation score used for comparison between samples. Mean Z-scores for *LINE-1* methylation were also calculated. Z-scores and clinical/genetic features (age, stage, high risk therapy, *MYCN* amplification, 1p loss, and outcome) were compared statistically in the STATISTICA 8.0 software (Statsoft Inc., Tulsa, OK, USA). We used Fischer’s exact test to compare categorical variables; Mann–Whitney *U*-test and Kruskal-Wallis one-way analysis of variance were used to analyze groups of continuous data; and correlations between continuous data were assessed using Spearman Rank Order correlations. Patients were divided into two groups based on whether their average Z-score was higher or lower than the median of the Z-score average for the methylated genes. The groups were compared by log-rank test; results were illustrated using Kaplan-Meier plots. Two patients with undetermined cause of death were excluded from these calculations.

## Abbreviations

DNA: Methylation in neuroblastoma.

## Competing interests

The authors declare that there are no financial or other interests that relate to the current manuscript.

## Author’s contributions

NK and JG performed the experiments; NK analyzed the data; PK, JJ and TM provided the clinical material and information; NK, CL, and JG designed the study, and NK and CL contributed to writing the manuscript. All authors read and approved the final manuscript.

## Pre-publication history

The pre-publication history for this paper can be accessed here:

http://www.biomedcentral.com/1471-2350/13/83/prepub

## Supplementary Material

Additional file 1 ** Table S1.** Methylation densities for hypermethylated genes in primary tumors. Click here for file

Additional file 2 ** Table S2. **Methylation densities in primary tumors for genes without promoter hypermethylation involvement. Click here for file

Additional file 3 ** Table S3. **Details of Pyrosequencing assays. Click here for file
